# Resistance exercise and nutritional interventions for augmenting sarcopenia outcomes in chronic kidney disease: a narrative review

**DOI:** 10.1002/jcsm.12791

**Published:** 2021-09-28

**Authors:** Hanaa Noor, Joanne Reid, Adrian Slee

**Affiliations:** ^1^ Division of Medicine University College London London UK; ^2^ Diaverum Holding AB Branch Riyadh Saudi Arabia; ^3^ School of Nursing and Midwifery Queen's University Belfast Belfast UK

**Keywords:** Chronic kidney disease (CKD), Sarcopenia, Intervention, Resistance exercise, Nutrition

## Abstract

Sarcopenia is an age‐related progressive muscle disease characterized by loss of muscle mass, muscle strength and physical performance with high prevalence in chronic kidney disease (CKD). CKD is associated with decreased muscle protein synthesis and muscle breakdown due to a number of factors including, the uremic inflammatory environment of the disease. CKD patients are highly sedentary and at risk of malnutrition which may exacerbate sarcopenia outcomes even further. Short and long‐term exercise and nutritional interventions have been studied and found to have some positive effects on sarcopenia measures in CKD. This narrative review summarized evidence between 2010 and 2020 of resistance exercise (RE) alone or combined with nutritional interventions for improving sarcopenia outcomes in CKD. Due to lack of CKD‐specific sarcopenia measures, the second European Working Group on Sarcopenia in Older People (EWGSOP2) definition has been used to guide the selection of the studies. The literature search identified 14 resistance exercise‐based studies and 5 nutrition plus RE interventional studies. Muscle strength outcomes were increased with longer intervention duration, intervention supervision, and high participant adherence. Data also suggested that CKD patients may require increased RE intensity and progressive loading to obtain detectable results in muscle mass. Unlike muscle strength and muscle mass, physical performance was readily improved by all types of exercise in long or short‐term interventions. Four studies used RE with high‐protein nutritional supplementation. These showed significant benefits on muscle strength and physical performance in dialysis patients while non‐significant results were found in muscle mass. More research is needed to confirm if a combination of RE and vitamin D supplementation could act synergistically to improve muscle strength in CKD. The current evidence on progressive RE for sarcopenia in CKD is encouraging; however, real‐life applications in clinical settings are still very limited. A multidisciplinary patient‐centred approach with regular follow‐up may be most beneficial due to the complexity of sarcopenia in CKD. Long‐term randomized control trials are needed to verify optimal RE prescription and explore safety and efficacy of other nutritional interventions in CKD.

## Introduction

Sarcopenia is a progressive muscle disease that has been recognized by the International Classification of Diseases (ICD‐10‐MC) in 2017.^1^ Primary sarcopenia due to old age is prevalent in 6–19% of those ≥60 years of age in the general population; a range that depends on the definition used.^2^ The most prominent and widely accepted definition has been proposed by the second European Working Group on Sarcopenia in Older People (EWGSOP2).^3^ Their definition encompasses three key features of sarcopenia including loss of muscle strength, muscle mass, and physical performance while highlighting validated diagnostic tools (*Table* 1).^3^ From a physiological perspective, sarcopenia is primarily caused by impaired muscle protein synthesis^4^ and resistance to anabolic stimuli such as protein intake and muscle contraction^5^; rather than increased muscle breakdown.^6^

**Table 1:** EWGSOP2 2018 operational definition and tools for measuring sarcopenia factors^3^

Operational definition of sarcopenia		
Probable sarcopenia: Criterion 1		
Confirmed sarcopenia: Criteria 1 and 2		
Severe sarcopenia: Criteria 1, 2 and 3		
Criterion 1	Criterion 2	Criterion 3
*Low muscle strength measured by:*	*Low muscle mass measured by:*	*Low physical performance measured by:*
Grip strength	Appendicular skeletal muscle mass (ASMM) by dual‐energy X‐ray absorptiometry (DXA) Or ASMM predicted by Bioelectrical impedance (BIA)	Gait speed
Chair stand test (sit‐to‐stand)	Whole‐body skeletal muscle mass (SMM)	Short physical performance battery (SPPB)
	Lumbar muscle cross‐sectional area by computed tomography (CT) or Magnetic resonance imaging (MRI)	Timed‐up‐and‐go‐test (TUG)
		400 m walk or long‐distance corridor walk

Primary sarcopenia, however, is confounded by chronic disease causing further musculoskeletal dysfunction.^3^ This is evident in the case of chronic kidney disease (CKD) with disease prevalence increasing with age.^7^, ^8^ The uremic inflammatory environment of CKD along with other comorbidities promote muscle catabolism leading to alterations of the nutritional status and body composition of affected persons.^9^, ^10^ In patients with CKD, sarcopenia is associated with disease progression^11^, ^12^ increased frailty,^13^, ^14^ mortality,^15^, ^16^ and decreased quality of life.^11^ Patients with end‐stage renal disease (ESRD) experience sarcopenia at higher levels from the general population with prevalence as high as 32.7–73.5% in haemodialysis and 25.6–44% in peritoneal dialysis.^17^, ^18^

The key therapeutic options of sarcopenia management include one or a combination of exercise, nutrition, and pharmacological interventions.^19^, ^20^, ^21^ A growing body of literature has reported sarcopenia specific benefits of exercise in CKD across the disease spectrum. Most recently, two systematic reviews concluded that regular RE and aerobic exercise (AE) are associated with improved health outcomes such as physical fitness, walking capacity, and cardiovascular health in CKD Stages 2–5, ESRD on dialysis therapy, and in kidney transplant.^22^, ^23^ A 2019 systematic review and meta‐analysis on the other hand highlighted that while both types of exercise showed association with improved physical performance in ESRD, regular RE showed more pronounced benefits on muscle mass and muscle strength especially for the trained muscles.^24^ These positive outcomes have been indicated in two earlier RE focused systematic reviews in non‐dialysis CKD Stages 3–5 and ESRD patients.^25^, ^26^ The authors concluded that RE, particularly progressively loaded training, can induce improvements in sarcopenia and health‐related quality of life.^25^, ^26^ However, available evidence is still not conclusive on optimal RE prescription especially in ESRD.^25^, ^27^, ^28^ This may be due to lack of effect (or propensity) in some studies hypothetically linked to hypercatabolic nature of the disease and lack of anabolic stimuli.

Apart from physical inactivity, malnutrition constitutes the highest concern in CKD patients who are reported to be prone to nutrient and energy stores wasting, exacerbating sarcopenia outcomes.^9^, ^29^ This is in part due to manifestations of systemic inflammation linked to body protein losses; a state of metabolic and nutritional derangements clinically known as protein‐energy wasting (PEW).^29^ In CKD Stages 2–5D, PEW is found to be prevalent in 11–54%, while 28–52% prevalent in transplanted patients.^9^ Causes include amongst others; uremia‐induced alterations such as low dietary intake, pro‐inflammatory environment, and high nutrients requirements leading to a hypercatabolic state.^30^ Despite common causes of malnutrition across the CKD spectrum, interventional nutritional strategies differ based on CKD stage.

According to the recently released Kidney Disease Outcomes Quality Initiative (KDOQI) 2020 clinical practice guidelines for nutrition in CKD, daily protein intake (DPI) recommendation varies according to physiological needs related to CKD stage.^31^ CKD 3–5 patients are prescribed low DPI (0.55 to 0.60 g/kg/day) aiming to postpone dialysis by reducing uremic clinical symptoms associated with protein metabolism.^31^ Because the dialysis treatment is characterized by loss of protein and increased muscle catabolism, patients treated with dialysis are prescribed high DPI (1.0–1.2 g/kg/day) to prevent PEW.^31^ Therefore, anabolic interventions using high‐protein oral nutritional supplementation (ONS) are often prescribed to support the nutritional status of dialysis patients.^20^ However, high‐protein ONS is not specifically indicated for sarcopenia management due to limited evidence.^20^, ^32^ A recent systematic review reported that high‐protein ONS resulted in little to no effect on lean body mass in 189 ESRD dialysis patients based on sub‐analysis of 5 randomized control trials (RCTs).^33^ In a selection of another four studies, mid‐arm muscle circumference (MAMC) was significantly improved in 216 ESRD dialysis patients post‐ONS; however, evidence was deemed to be of low certainty.^33^

In comparison with CKD, nutritional interventions for sarcopenia management are widely studied in the general older population ^34^, ^35^. A key systematic review of 37 RCTs of ONS in older people reported favourable outcomes in muscle strength and muscle mass especially with very high‐protein doses (>20 g/days).^32^ Interestingly, when combined with exercise, limited impact on muscle function was found.^32^ Nonetheless, the systematic review indicated that the majority of subjects included were healthy older adults with low risk of malnutrition at baseline, which could explain limited intervention effects.^32^

Of note, there is emerging evidence that supports the use of other nutrients besides protein to attenuate sarcopenia in older people. A 2020 review on primary sarcopenia described promising effects of various nutritional interventions including protein, amino acids, vitamin D, omega‐3 fatty acids, and magnesium amongst others.^34^ This data, interpreted in the light of CKD pathophysiology and added catabolic effects of haemodialysis in ESRD, suggest that adding nutritional interventions to RE may show similar or higher benefits in CKD than in general well‐nourished populations. Therefore, the first aim of this review is to summarize the most recent evidence on RE‐based interventions for sarcopenia in CKD. The second aim is to explore availability of evidence to support whether a combination of RE and nutrition supplementation is warranted to improve sarcopenia outcomes in CKD.

## Search strategy and criteria

PubMed, Cochrane, Science Direct, and Google Scholar databases were searched using the following keywords alone or in combination of others that are related to the topic, namely, sarcopenia and CKD and/or ESRD, muscle strength and CKD and/or ESRD, muscle mass and CKD and/or ESRD, physical performance/function and CKD and/or ESRD, and exercise interventions (aerobic and resistance) in CKD and/or ESRD. Different nutritional interventions in sarcopenia reviewed by Cruz‐Jentoft *et al*.^34^ were searched in combination with RE in CKD including protein and amino acids, omega‐3 fatty acids, vitamin D, vitamin C and E, selenium, magnesium, phytonutrients/polyphenols, dairy products, and probiotics.

The search of clinical studies was confined to CKD and ESRD interventional studies between 2010 and 2020 and limited to English language and human trials only. Because there is no current sarcopenia definition and cut‐offs specific to CKD^36^; the European consensus on definition and diagnosis of sarcopenia in older people was used to guide the selection of the studies.^3^ Accordingly, this review includes studies with at least one primary or secondary sarcopenia outcome measures based on tests indicated in *Table* 1. Studies that used different measures than those listed in *Table* 1 were excluded to reduce heterogeneity. An emphasis was made on RCTs where available.

## Resistance exercise interventions

The RE has been extensively studied on its direct and indirect effects on muscle and protein turnover. In the ageing population, RE has been found to enhance neuromuscular function and motor unit activation patterns leading to enhanced strength and power.^37^ In addition, the repetition of training sessions typically stimulates net protein synthesis through the activation of mammalian target of rapamycin (mTOR) signalling pathway, increasing muscle mass growth.^37^ It is also understood that RE may increase anabolic hormone production such as testosterone, growth hormones, and insulin‐like growth factor 1 (IGF‐1).^38^ These effects are particularly important in CKD considering inflammation as a modulating factor, as it may be that any potential muscle gains are hampered by background inflammatory signals.

A recent study by Hangelbroek *et al*.^39^ investigated the effects of a 24 week progressive RE programme in older frail and pre‐frail adults. The study observed a negative association between the plasma levels of pro‐inflammatory cytokines (in particular, tumour necrosis factor alpha; TNF‐α) and the adaptive response to RE, in that strength gains were lower in those participants with higher background inflammation.^39^ Nevertheless, exercise and resistance training are associated with significant anti‐inflammatory effects, although this has not been found in all studies.^40^ Interestingly, a recent study by Sardeli *et al*.^41^ observed that RE reduced inflammation in older adults, in particular reductions in inflammatory marker C‐reactive protein (CRP) and TNF‐α only in higher number of exercises (>8), higher weekly frequency (3×/week) and longer durations (>12 weeks). Therefore, adjusting to have higher RE volume, intensity, and duration may potentially counteract the catabolic effects of inflammation in CKD.

In 157 older ESRD patients, a recent study found that 24 weeks of RE (3×/week) significantly decreased inflammation [decreases in TNF‐α and interleukin (IL)‐6], increased anti‐inflammatory molecule IL‐10, improved iron bioavailability (*P* < 0.0001) and reduced hepcidin (*P* < 0.0001).^42^ Note, of interest, the study was designed so that participants performed RE 1 hour before haemodialysis which may have yielded a more robust anti‐inflammatory effect because haemodialysis treatment modality in itself induces catabolism.^42^ A shorter 12 week intradialytic (i.e. during haemodialysis) RE also produced significant reductions in CRP in a randomized controlled model (*P* < 0.05).^43^ In non‐dialysis CKD Stages 3–4, an older study which investigated using RE (3×/week for 12 weeks); found significant decreases in inflammation (CRP and IL‐6) and increased muscle mass and strength.^44^ The study also confirmed an inverse association between changes in IL‐6 and muscle fibre type size (type I and II) and muscle strength.^44^

Given the complex interplay between inflammation and physical inactivity, KDOQI recommends CKD patients to engage in at least 30 min moderately intense physical activity in most or all days of the week to mitigate risk of cardiovascular disease associated with kidney failure.^45^ No similar exercise recommendations are available for sarcopenia management in CKD and ESRD.^46^, ^47^ In reality, CKD and ESRD patients are far less likely to meet KDOQI's recommendations and are much more sedentary when compared with healthy sedentary individuals or patients with other chronic diseases.^48^, ^49^ Commonly cited reasons of decreased physical activity in CKD and ESRD include anaemia, poor nutritional status, bone disease, comorbid conditions, and dialysis treatment related fatigue.^48^, ^49^

We have identified 14 studies that developed RE‐based interventions according to the search criteria; 2 of which were based on the Renal Exercise study (RENEXC) with each reporting different sarcopenia outcomes.^50^, ^51^ Out of 14 trials, 8 prescribed RE in ESRD on dialysis^43^, ^52^, ^53^, ^54^, ^55^, ^56^, ^57^, ^58^ while 5 included non‐dialysis CKD patients' Stages 3–5,^50^, ^51^, ^59^, ^60^, ^61^ and only 1 study had transplanted patients.^62^ Sample size varied substantially between studies ranging from 19 to 151 participants with median sample size of 46. Duration of intervention ranged between 3 and 12 months with median duration of 3.5 months. Eight studies included patients ≥65 years^50^, ^51^, ^52^, ^53^, ^57^, ^59^, ^60^, ^61^ while six examined intervention effects in younger population.^54^, ^55^, ^56^, ^58^, ^62^ Exercise intensity was monitored using Borg's rating of perceived exertion (RPE)^63^ or based on one‐repetition maximum (1‐RM)^64^ across the studies. Description of studies and summary of results are presented in Appendix 0.

### Muscle strength

Muscle strength was measured using grip strength (GS) and/or sit‐to‐stand (STS) tests. Duration of intervention, intervention supervision, and participants adherence appear to be the three key factors modulating the outcome in muscle strength. Long‐term 12 month intervention was reported in four studies; one included ESRD haemodialysis patients (non‐RCT)^52^ and 3 included CKD non‐dialysis patients Stages 3–5 (RCTs).^51^, ^59^, ^60^ When compared with baseline, a statistically significant improvement in STS test observed at 6 and 12 months in all haemodialysis patients participating in a structured supervised RE plus endurance training (ET) programme.^52^ However, the size of the observed muscle strength improvement was significantly impacted by adherence rate. In fact, when patients were further analysed according to adherence; high adherence (HA) group showed statistically significant strength improvements through RE in all eight exercises (*P* ≤ 0.001) while medium adherence (MA) group improved significantly in only two exercises (leg extensor *P* = 0.002, abductor *P* < 0.001).^52^

In CKD Stages 3–5 non‐dialysis 12 months interventions, a similar trend towards muscle strength improvement was found; albeit, results were inconsistent. To demonstrate, Hiraki *et al*.^59^ home‐based RE plus AE induced a 17.0 ± 16.1% significant improvement in GS, while Hellberg *et al*.^51^ reported only significant positive change in STS but not GS after 12 months of self‐administered RE plus ET. This result was consistent with no change observed in GS after 8 weeks of supervised exercise and lifestyle intervention followed by 10 months of home‐based exercise programme.^65^

Lack of timely follow‐up and direct supervision are possible explanations of the inconsistent results observed in non‐dialysis interventions. RE prescription for non‐dialysis CKD stage 3–5 is often self‐administered without direct supervision and follow‐up held every 2–3 months during routine clinic visits..^51^, ^59^, ^60^ In contrast, RE for dialysis patients is usually held in clinic with direct support and feedback from healthcare team and fellow exercising patients possibly increasing motivation and adherence.^52^ Nonetheless, a simple follow‐up scheme had shown potential to overcome low adherence rates in a similar population group. A home‐based 12 week RE intervention in kidney transplanted patients demonstrated that weekly follow‐up and discussion telephone calls increased motivation and RE adherence. This had a clear effect on muscle strength where significant improvements in STS test in the RE group were reported.^62^

Short‐term RE interventions measuring muscle strength ranged between 3–4 months were prescribed in 7 studies; 6 in ESRD haemodialysis (4 RCTs; 2 non‐RCTs)^43^, ^54^, ^55^, ^56^, ^57^, ^58^ and 1 in CKD stages 3–4 non‐dialysis patients (RCT).^61^ In haemodialysis interventions, muscle strength outcome was clearly confounded by short duration resulting in non‐significant improvements in GS and STS (non‐RCT; RCT).^56^, ^58^ However, increasing exercise frequency in short‐term interventions led to increased efficacy of intervention in ESRD haemodialysis patients. To illustrate, a 12 week RCT prescribed RE twice weekly during haemodialysis reported significant improvement of 9.82% in GS^54^ while higher RE prescription of three times per week of a similar RCT intervention induced a 23.54% improvement in GS.^43^ Interestingly, adding 4 weeks to the conventional 12 week interventions; improved overall muscle strength and produced significant improvements in GS in a loaded cycling intervention even though upper body muscles were not trained (non‐RCT).^57^

These findings may support the hypothesis that higher dosing of exercise regimen might be needed to overcome effects of both the ageing‐related anabolic resistance and catabolic state related to both renal failure as a disease and haemodialysis being a catabolic intervention itself.^5^, ^10^ In CKD Stages 3–4 non‐dialysis patients, combining AE and RE may assist in overcoming the confounding effect of short intervention on muscle strength. An RCT demonstrated this by developing a renal rehabilitation programme combining AE and RE, which resulted in a significant change in STS (+29%) in a 12 week intervention consisting of only twice weekly training sessions.^61^

### Muscle mass

Muscle mass was measured by either whole‐body skeletal muscle mass (SMM) and/or appendicular skeletal muscle mass (ASMM) by dual‐energy X‐ray absorptiometry (DXA) and/or bioelectrical impedance (BIA). Only 7 out of 14 studies measured muscle mass as an outcome (5 RCTs; 2 non‐RCTs).^43^, ^50^, ^53^, ^55^, ^56^, ^57^, ^58^ Unlike muscle strength, duration of intervention was not a highly predictive factor in muscle mass. Rather exercise intensity and size of progressive weight loading played a major role. Desai *et al*. (non‐RCT)^57^ reported non‐significant changes in ASMM and SMM measured by BIA after a 4 month loaded cycling intervention in ESRD haemodialysis patients. Lack of effect could be related to low non‐progressive load during intervention, although exercise intensity was aimed to reach 13–15 (moderate) on the RPE scale.^57^ Alternatively, a shorter RCT 12 week intervention in ESRD haemodialysis patients induced positive increases in ASMM measured by MRI through continuous adjustments of repetitions, intensity (80% 1‐RM) and progressively increased load.^58^

Similarly, a significant increase in SMM by BIA in a 12 week intervention was reported using progressively increased weight to ankles and increasing tensile strength of elastic bands used to train upper body muscles (non‐RCT).^56^ Significant findings from a longer 6 month intervention of progressive RE in ESRD on haemodialysis also support the effectiveness of continuous incremental adjustment of both exercise intensity and weight to increase muscle mass (SMM by DXA + 4.2 ± 5.6%; ASMM by DXA + 5.0 ± 7.6%—*P* < 0.001; RCT).^53^ However, evidence is inconsistent as no change in SMM by BIA was reported after a similarly structured progressive RE intervention held in ESRD haemodialysis patients.^43^

Noteworthy, all above cited interventions prescribed exercise during haemodialysis sessions.^43^, ^50^, ^53^, ^55^, ^56^, ^57^, ^58^ Reasons include characteristics of the dialysis population, perceived efficiency and efficacy, convenience, and cost. Older dialysis populations are reported to be less likely to perform training on their own either due to fatigue after haemodialysis sessions or due to safety concerns.^43^, ^50^, ^53^, ^55^, ^56^, ^57^, ^58^ By design, having an intradialytic exercise programme is more efficient as it utilizes time spent on dialysis machines counteracting effects of haemodialysis being mostly a sedentary intervention. Other reasons include perceived benefits of group exercises to the motivation and compliance of patients.^43^, ^50^, ^53^, ^55^, ^56^, ^57^, ^58^ Song *et al*.^56^ further explains that group exercises are associated with higher interest and positive emotional effects when compared with individual exercises, which inevitably boosts motivation and enhances compliance. Additionally, intradialytic interventions are reported to have lower costs than individualized outside dialysis sessions where an established infrastructure and dedicated trainers are possibly needed.^43^, ^50^, ^53^, ^55^, ^56^, ^57^, ^58^ Convenience was another factor cited by the studies to justify an intradialytic‐based intervention. These studies propose to incorporate intradialytic exercise programmes within routine care of patients with kidney failure making it more applicable and accessible to different haemodialysis settings.^43^, ^50^, ^53^, ^55^, ^56^, ^57^, ^58^ Lastly, intradialytic exercise programmes are natural setups for direct supervision where healthcare staff and exercise physiologists can ensure proper technique, boost motivation, and monitor training progress and safety.^43^, ^50^, ^53^, ^55^, ^56^, ^57^, ^58^

All above factors might have collectively increased the likelihood of a positive outcome on muscle mass in ESRD intradialytic interventions. This is unlike interventions in non‐dialysis Stages 3–5 CKD where cost, time, and direct supervision were potential barriers for detectable significant outcomes. To illustrate, contrary to authors' hypothesis, the RENEXC non‐supervised intervention of RE plus ET in non‐dialysis Stages 3–5 CKD showed no significant increase in both ASMM and SMM by DXA after 12 months (RCT).^50^ No direct supervision meant that collected data on training adherence, intensity, and time were based on self‐reported participants diaries, which may have introduced bias and error through overestimation or inaccurate reporting.^50^ In contrast, a much shorter intervention (12 weeks) had significant increase in ASMM by DXA post‐intervention in ESRD haemodialysis utilizing direct supervision by a clinical exercise physiologist.^55^ Therefore, it can be suggested that future research focuses on emulating intradialytic interventions that includes group supervised exercises into non‐dialysis CKD intervention settings to obtain best possible outcome.

### Physical performance

Physical performance was determined by either gait speed and/or short physical performance battery (SPPB), timed‐up‐and‐go‐test (TUG), and/or 600 m walk test (6‐MWT). Only 9 out of 14 studies measured physical performance as an outcome (7 RCTs and 2 non‐RCTs).^43^, ^51^, ^52^, ^53^, ^55^, ^57^, ^58^, ^60^, ^61^ Unlike muscle strength and muscle mass, physical performance was readily improved by all types of exercise in long‐term or short‐term interventions. In fact, the largest study included in this review showed that all 151 non‐dialysis Stages 3–5 CKD participants randomized to either balance training plus ET or RE plus ET had significant improvements in 6‐MWT when compared with their baseline (RCT).^51^ No evidence was found to support superiority of one exercise programme over the other in improving physical performance.^51^

Furthermore, long‐term 12 months studies in non‐dialysis Stages 3–4 CKD and ESRD on haemodialysis showed significant improvement in 6‐MWT post intervention, while either improving TUG at 12 months in haemodialysis (non‐RCT)^52^ or preventing TUG decline observed in non‐dialysis control group (RCT).^60^ Furthermore, 4–6 months interventions of progressive RE in ESRD on haemodialysis showed a significant positive outcome on SPPB (+21.1%; *P* < 0.05; RCT)^53^ and equally in 6‐MWT (non‐RCT).^57^ Shorter duration 12 weeks interventions demonstrated significant positive improvement in 6‐MWT in both haemodialysis (RCTs)^43^, ^55^ and non‐dialysis CKD Stages 3–4 patients, but no change was detected in gait speed (RCT).^61^ Kirkman *et al*.^58^ reported positive changes in 6‐MWT and TUG in an RCT; however, effect size did not detect significance post 12 weeks ESRD on haemodialysis patients. This might be due to small sample size of 19 participants randomized to either RE or sham exercise.^58^

## Resistance exercise plus nutritional interventions

Protein and vitamin D supplementation were the only nutrients found to have been investigated with RE to target sarcopenia in CKD. As a general rule, high‐protein supplementation is prescribed when needed for ESRD dialysis patients considering their high daily protein requirement as per KDOQI's clinical practice guidelines.^31^ On the other hand, vitamin D supplementation is recommended in all stages of CKD to prevent insufficiency and deficiency.^31^ This is particularly important because vitamin D deficiency is common in CKD patients due to loss of kidney function and impaired vitamin D activation.^66^, ^67^

We have identified only one study that prescribed RE intervention with vitamin D (non‐RCT)^68^ and four studies prescribing RE with high‐protein ONS; two of which are RCTs^69^, ^70^ and two are non‐RCTs.^71^, ^72^ RE plus vitamin D examined effects in non‐dialysis CKD Stage 4 patients^68^ while RE plus ONS were prescribed in dialysis patients only because high‐protein ONS is contraindicated in non‐dialysis patients.^69^, ^70^, ^71^, ^72^ Mean sample size of included studies is 32 (±6.7 *SD*) while median duration of intervention is 12 weeks (range 12–24). All five studies investigated outcome in population <65 years of age.^68^, ^69^, ^70^, ^71^, ^72^ Heterogeneity is evident in both the type of exercise and the nutrition interventions specifically protein content of prescribed ONS (median 39.2 g/week; range 28.4–199.2).^69^, ^70^, ^71^, ^72^ Description of studies and summary of results are available in Appendix 0.

### Resistance exercise plus high‐protein supplementation

Dong *et al*.^69^ was the only study reporting muscle mass as an outcome. Two cans of high‐calorie high‐protein ONS providing 66.4 g of protein were administered three times weekly for 6 months. RE was progressively loaded, and intensity was adjusted two times during the study to reach 70% 1‐RM. No significant change between ONS only and ONS plus RE was detected in SMM and ASMM by DXA.^69^ There were two speculative reasons suggested by the authors for lack of effect on muscle mass. First, participants were younger than the general dialysis population meaning that potentially any change in muscle mass could not be detected as much as in older patients with clear muscle wasting. Additionally, exercise intensity and duration may have been inadequate to induce significant changes in muscle mass.^69^

Contrary to muscle mass, interventions targeting muscle strength and physical performance showed advantageous outcomes in dialysis patients younger than 65 years of age. Physiologically speaking, the absence of resistance to anabolic stimuli in young patients seemed to allow for detectable improvements even with short‐term high‐protein ONS interventions^5^, ^73^ To illustrate, the AVANTE‐HEMO study conducted three‐armed intervention of ONS only, ONS plus AE, and ONS plus RE for 12 weeks in ESRD haemodialysis patients with mean age of 29 ± 9.3.^72^ GS was significantly improved post intervention in all groups (*P* < 0.05) while STS test was significantly improved in exercise groups only (*P* < 0.05).^72^ Effect size showed that ONS plus RE had higher effects on GS followed by ONS plus AE and then ONS group. Alternatively, AE plus ONS had the highest effect size on STS followed by RE plus ONS and ONS group.^72^

Similarly, all groups showed significant improvements in physical performance measured by TUG and 6‐MWT (*P* < 0.05) with AE plus ONS having the biggest effect size.^72^ It can therefore be assumed that adding any type of exercise to ONS would be more beneficial to muscle strength and physical performance than ONS alone. However, these positive outcomes may be directly affected by the age of participants included being younger than general dialysis populations.^7^ Therefore, one could argue the limited reproducibility of these results in older frail sarcopenic patients with limited mobility and lower ability to complete prescribed exercise protocols.^10^, ^48^, ^49^ Significant improvement in GS was also observed in ONS only and ONS plus RE interventions in an earlier study carried by the same investigators at the same haemodialysis clinic.^71^ However, effect size between interventions was not reported.^71^ Accordingly, more studies are needed to confirm the above findings.

Molsted *et al*.^70^ attempted to test the effects on muscle strength using timed protein and non‐protein supplements in combination to RE. For 16 weeks, ESRD haemodialysis and peritoneal dialysis participants engaged in supervised progressive RE outside of dialysis and received equal energy supply as base. The RE plus protein group consumed 250 kcal (9.4 g protein, 25 g carbohydrates, 12.5 g lipids) three times per week within 2 h on either side of the exercise session.^70^ RE plus non‐protein group consumed a similar calorie intake of 250 kcal with energy supplied by carbohydrates (2.4 g) and lipids only (27.3 g).^70^ Contrary to authors' hypothesis, no difference was found in STS test between groups, and significant improvement was similarly observed in all participants when compared with their baseline.^70^

It is worth noting that the study did not take into account possible additional benefit of the added energy supply in their analysis making it unclear whether the additional energy equalized outcome between groups. In other words, no direct association can be drawn between protein supplementation with RE and positive effects on muscle strength because study the design did not include a control group. Furthermore, there was no record of total DPI (grams per day).^70^ Lack of difference between groups may also be due to a lower protein dose in the protein supplementation group than that was actually needed to overcome both age‐related and disease‐related muscle strength losses.^35^, ^70^ In fact, according to the PROT‐AGE Study Group, older healthy adults require more dietary protein intake to maintain or regain muscle when compared with younger population.^74^ An anabolic protein threshold has been consequently identified as 25–30 g protein per meal.^74^ This threshold is notably higher than Molsted *et al*.^70^ total of 18.8 g protein prescribed for dialysis patients in the RE plus protein group. Therefore, a higher protein dose may be warranted to induce detectable results in muscle strength.

### Resistance exercise plus vitamin D supplementation

Olvera‐Soto *et al*.^68^ conducted the only interventional study to use a combination of cholecalciferol (vitamin D) supplementation and RE in non‐dialysis CKD Stage 4 patients to target muscle mass and muscle strength. No similar studies were found in dialysis patients. The 12 week intervention included an individualized supplementation protocol of vitamin D according to patient's baseline serum level.^68^ The RE programme was self‐administered focusing mostly on upper body muscles (5/6 exercises). Although adherence rates were relatively high (77% in RE, 96.2% in vitamin D), no significant changes were reported in SMM by BIA while a trend towards muscle mass increase within intervention group was observed.^68^ This might be due to lack of progressive loading and lack of monitoring of exercise intensity needed to induce detectable change in whole‐body muscle mass. Alternatively, muscle strength measured by GS of both right and left hands were significantly improved (*P* < 0.05) together with vitamin D serum levels (*P* < 0.05). Conversely, the control group had a decrease in both serum vitamin D and GS.^68^ More research is needed to confirm if a combination of RE and vitamin D supplementation could act synergistically to improve muscle strength in CKD.

Of note, the replicability of similar adherence rates in real life settings might not be achievable. Allocation to either intervention or control groups was based on patient's interest. In other words, participants in the intervention group inherently had high motivation to complete the prescribed intervention resulting in high adherence rates.^68^ Because study participants were relatively young with median age of 48 (range 36–52), future studies are needed to determine replicability of these results in an older sarcopenic CKD population. Moreover, an interventional study reported highest improvement in physical performance after vitamin D supplementation in severely deficient peritoneal dialysis and non‐dialysis CKD patients.^75^ Based on this evidence, future studies should consider statistical analysis of sarcopenia outcomes based on stratification of vitamin D levels at baseline. Additionally, including vitamin D only and RE only control groups could provide new insights based on effect size comparison of separate vs. combined interventions.

## Limitations

Even though this review aimed to summarize evidence of RE‐based interventions in sarcopenic CKD patients, only 2 out of 19 studies had in fact reported sarcopenia prevalence at baseline.^43^, ^50^ The first study by Dong *et al*.^43^ was the only study that used sarcopenia diagnosis as an inclusion/exclusion criterion using the Asia Working Group for Sarcopenia (AWGS) definition, which is not CKD specific.^76^ While the second study by Zhou *et al*.^50^ only compared sarcopenia prevalence pre and post intervention without using sarcopenia diagnosis to determine eligibility. It was therefore not possible for this review to focus solely on sarcopenic patients. Subsequently, this review included interventions with at least one sarcopenia measured outcome of muscle mass, muscle strength, or physical performance regardless of sarcopenia diagnosis at baseline.

This review was also limited by the heterogeneity of prescribed interventions and protocols in the studies, which inevitably impacted the comparability of outcomes. This includes lack of a good control group in the majority of the studies, limiting the review's analysis in some instances to discuss efficacy based on intragroup differences. In addition, RE dose, intensity, and duration varied widely amongst studies as well as ONS composition, frequency, and duration. In the same vein, assessment tools of sarcopenia outcomes were highly variable with the largest inconsistency observed in muscle mass. This is evident in measurements derived from BIA compared with more accurate measurements produced by DXA.^77^ BIA estimates muscle mass from whole body electrical resistance, which means it is likely that BIA may potentially overestimate muscle mass in dialysis patients experiencing volume expansion.^78^

Additionally, the majority of the studies available targeted prevalent haemodialysis patients (>3 months dialysis) with limited evidence available for interventions employed to support incident dialysis patients that are new to dialysis, peritoneal dialysis, or kidney transplanted patients.^43^, ^52^, ^53^, ^54^, ^55^, ^56^, ^57^, ^58^, ^69^, ^70^, ^72^ Consequently, it was not possible to evaluate the impact of dialysis vintage on exercise and nutritional interventions in this review. Most studies were also plagued by a short duration and a small number of participants, reducing the generalizability of the observed results.

## Conclusions and future directions

The current evidence on progressive RE in CKD is encouraging although real‐life applications in clinical settings are still very limited.^46^, ^47^, ^79^ Sustainable long‐term interventions require commitment not only from patients and their caregivers but more importantly, they require commitment from the healthcare team.^79^, ^80^, ^81^ Healthcare professionals act as gatekeepers for health information^82^; thus, staff education on the importance of sarcopenia screening, its debilitating effects, and possible interventions should be a priority. Additionally, behavioural change strategies and motivational interviewing training are important skills for healthcare professionals to be able to assist patients in sustaining motivation and increasing adherence.^82^

Based on evidence presented in this review, sarcopenia diagnosis and severity assessment at baseline should be considered in order to tailor exercise interventions. Sarcopenic patients may have reduced mobility, thus, conditioning or rehabilitative exercises should be considered prior to prescribing interventions to ensure safety and avoid potential injuries.^32^ In addition, correcting malnutrition status and nutrient deficiencies in CKD patients should be prioritized because data suggest low exercise efficacy in malnourished patients.^29^, ^34^

**Figure 1:**
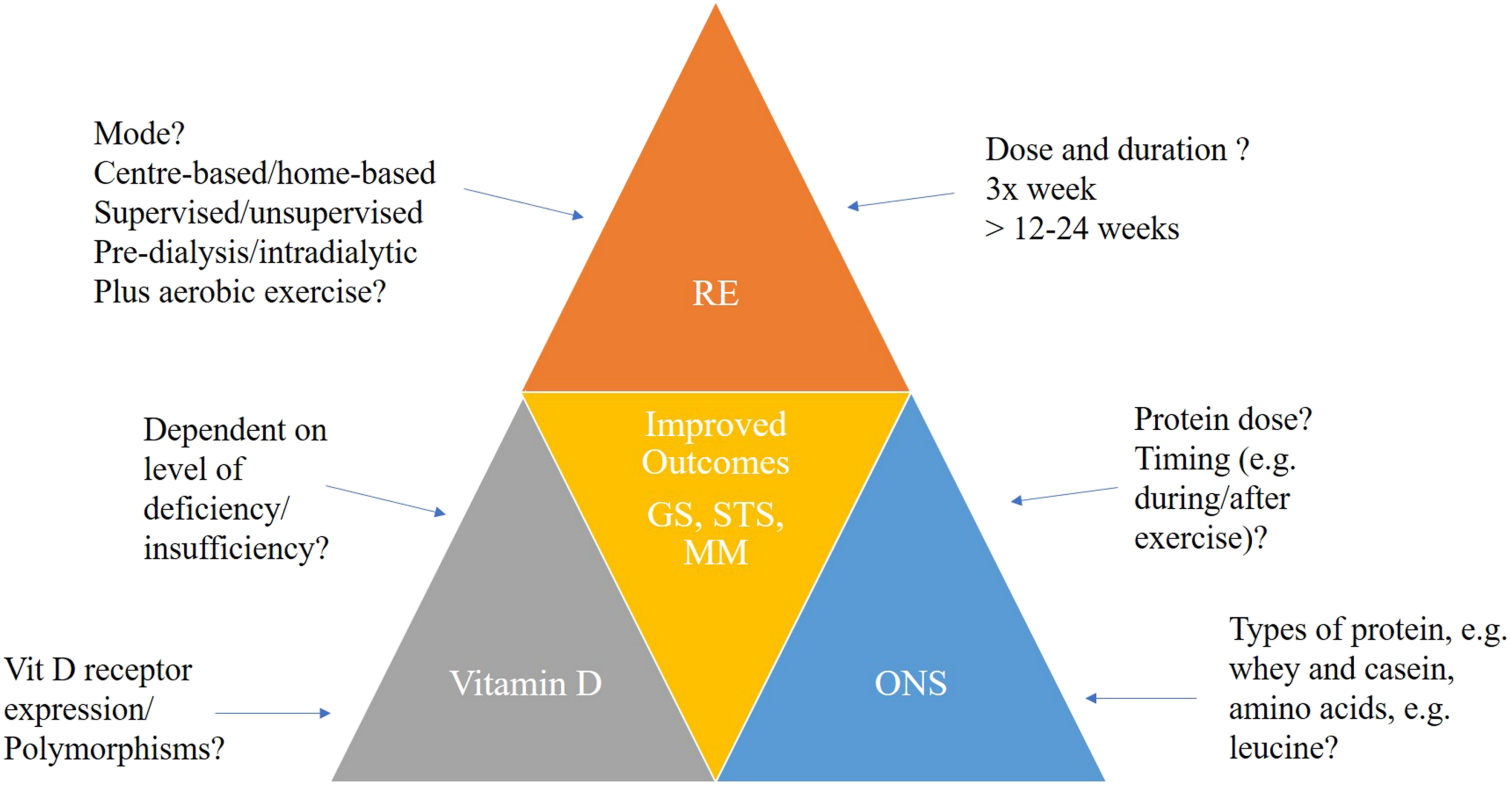
Possible interactions and consideration between RE, ONS, and vitamin D on outcomes for CKD patients. GS, grip strength; MM, muscle mass; ONS, oral nutritional supplementation; RE, resistance exercise; STS, sit‐to‐stand test.

*Figure* 1 illustrates proposed future directions based on possible interactions between factors reviewed in this paper including RE, vitamin D and ONS to improve sarcopenia outcomes.

To conclude, sarcopenia is associated with debilitating outcomes in CKD due to the catabolic nature of CKD coupled with anabolic resistance of the ageing muscle. Electronic databases search identified 14 RE‐based studies and 5 RE plus nutritional interventions studies. Data suggest that RE with or without nutritional interventions is a valuable and underutilized tool for improving muscle mass, muscle strength, and physical function in CKD patients. Adding high‐protein ONS or vitamin D to RE have possible added benefits; however, data are still limited. In a clinical setting, a multidisciplinary individualized approach to supporting patients with sarcopenia is possibly the most beneficial. Finally, long‐term RCTs are needed to create CKD‐specific sarcopenia definition and cut‐offs, verify optimal RE prescription, and explore safety and efficacy of other nutritional interventions that were previously investigated in the general older population.

## Conflict of interest

The authors declare that they have no relevant conflicts of interest.

## Funding

This work is supported and funded by Diaverum Holding AB Branch.

## Appendix 1 Clinical studies of resistance exercise‐based interventions and their effects on sarcopenia outcomes in CKD

**Table:**

A structured exercise programme during haemodialysis for patients with chronic kidney disease: clinical benefit and long‐term adherence				
Author, year	Anding *et al*., 2015^52^	Duration of intervention	12 months	
Participants CKD stage Age (years)	ESRD on haemodialysis 63.2 ± 16.3 (1) High adherence (HA): 19 HA, >80% of 104 training sessions within 12 months (2) Moderate adherence (MA): 12 MA, 60–80% of 104 training sessions within 12 months (3) Low adherence group (LA): 15 LA, <60% of 104 training sessions within 12 months	Sarcopenia outcomes		
		Muscle mass	Muscle strength	Physical performance
		NA	At 6 months (HA, MA): ↑STS** At 12 months (HA, MA): ↑STS***	At 6 months (HA, MA): ↓TUG** ↑6MWT (NS) At 12 months (HA, MA): ↓TUG*** ↑6MWT***
Intervention description				
Structured physical exercise programme (SPEP) supervised by exercise specialist: 2×/week of RE + ET for 60 min during first 2 h of haemodialysis. Intensity continuously adjusted to improvements of performance testing. Start: 5 min warm‐up Endurance training: Bed‐cycle ergometers positioned in front of patients' chairs. Participants continue until muscular fatigue. Dynamic resistance training: Weights and elastic bands used. Training of 8 muscle groups with an individual target repetition rate (R) of exercises in 2 sets of 1 min each with 1 min break. The target repetition rate was derived from the maximal repetition rate (MRR) in a maximum strength test for all 8 muscle groups; patients were asked to perform as many repetitions as possible in 1 min. Month 1: goal to achieve 50% MRR, Months 2 + 3: 65% MRR, Months 5 + 4: 70% MRR. After Month 5: MRR test repeated to set new one. Months 6–10: as 1–5 based on new MRR.				

Effect of intra‐dialytic, low‐intensity strength training on functional capacity in adult haemodialysis patients: a randomized pilot trial				
Author, year	Chen *et al*., 2010^53^	Duration of intervention	6 months (48 exercise sessions)	
Participants CKD stage Age (years)	ESRD on haemodialysis 69 ± 13 E1: 22 E2: 22	Sarcopenia outcomes		
		Muscle mass	Muscle strength	Physical performance
		↑SMM by DXA (4.2 ± 5.6%)*** ↑ASMM by DXA (5.0 ± 7.6%)***	NA	↑SPPB (21.1%)*
Intervention description				
2×/week during the 2nd hour of haemodialysis Start: 5 min warm‐up and end with 5 min cool‐down E1: Supervised progressive resistance exercise 2 sets/8 repetitions per exercise with 1–2 min rest between sets. Conditioning: first 8 exercise sessions no or little weight is used and progressed based on participants' ability to complete 2 sets/8 repetitions with proper form and rate of perceived exertion (RPE) of 2–4 (easy to somewhat easy). Next; multiple lower body seated exercises using ankle weights increasing in half‐pound increments from 0.5 to 20 lbs. Seated pelvic tilt without using free weights. Moderate intensity (somewhat hard) corresponding 6 on RPE scale. E2: Attention‐control: Stretching exercises with light resistance bands Exercises include ankles flexion, rotation, calf, hamstring and inner thigh stretch. Exercises were done in the semi recumbent position, held for 20–30 s and repeated ×2.				

A pilot study investigating the effect of pedalling exercise during dialysis on 6 min walking test and hand grip and pinch strength				
Author, year	Desai *et al*., 2019 ^57^	Duration of intervention	4 months	
Participants CKD stage Age (years)	ESRD on haemodialysis 64.0 ± 16.6 E: 13 C: 21	Sarcopenia outcomes		
		Muscle mass	Muscle strength	Physical performance
		↑SMM by BIA (NS) ↓ASMM by BIA (NS)	↑Grip strength**	↑6‐MWT*
Intervention description				
3×/week during haemodialysis E: Progressive sub‐maximal individualized cycling exercise with loading Exercise used bed‐cycle ergometers positioned in front of the patient's dialysis chair with exercise intensity monitored every 5 min. Warm‐up: low‐load aerobic cycling at an intensity of 8–9 on RPE scale. Conditioning: cycling with aim of 13–15 RPE (moderate intensity). Cooling down: light cycling with no load or resistance at an intensity of 8–9 RPE scale. C: Control group Received routine haemodialysis care.				

Effects of intradialytic resistance exercise on systemic inflammation in maintenance haemodialysis patients with sarcopenia: a randomized controlled trial				
Author, year	Dong *et al*., 2019 ^43^	Duration of intervention	12 weeks	
Participants CKD stage Age (years)	ESRD on haemodialysis 60 (43–68) E: 21 C: 20	Sarcopenia outcomes		
		Muscle mass	Muscle strength	Physical performance
		↔SMM by BIA (NS)	↑Grip strength (23.54%)**	↑6‐MWT (15.94%)*
Intervention description				
3×/week during haemodialysis E: progressive resistance exercise Start: 5 min warm‐up. 10 sets/10 repetitions of all exercises maximally maintained for 3–5 s per cycle and then release completing 1–2 h of RE during haemodialysis. 1st week: low intensity RE with no weights using quadriceps training board for assistance. 2–12 weeks: moderate to high intensity adding + 0.5 kg (single foot)/week to ankle to reach + 5 kg maximum Progression was done according to patient's tolerance with the angle of the training board reduced gradually (150°–90°) until it was removed. Upper limb exercises: non‐treated hand with elastic resistance ball. C: Control group Received routine haemodialysis care.				

Aerobic or resistance training and pulse wave velocity in kidney transplant recipients: a 12 week pilot randomized controlled trial [the Exercise in Renal Transplant (ExeRT) Trial]				
Author, year	Greenwood, *et al*., 2015^62^	Duration of intervention	12 weeks	
Participants CKD stage Age (years)	Kidney transplanted 54 ± 10.6 RE:13 AE:13 UC:20	Sarcopenia outcomes		
		Muscle mass	Muscle strength	Physical performance
		NA	↑STS* (within RE and within AE) ↑STS (RE/UC)** ↑STS (AE/UC) (NS)	NA
Intervention description				
2×/week free supervised structured aerobic exercise classes in a gym or hospital setting and 1×/week home‐based resistance exercise. Prior to start: all received 40 min individual behaviour modification session to discuss exercise and personal goals using motivational interviewing. Participants were instructed how to use an RPE scale to rate perception of effort at the prescribed exercise intensity RE: Home‐based resistance exercise 60 min once per week Resistance bands and ankle weights were provided. *Prescription:* warm‐up and cool‐down period of 5 min on a stationary exercise cycle, RPE of 11, followed by gentle stretching. RE of high‐intensity at 80% one‐repetition maximum, the maximum amount of weight can be lifted or pressed once but not twice, for upper and lower body muscle groups (bench press, latissimus pull down, bicep curl, triceps pull down, leg press, knee extension, hamstring curl, and calf raises). Duration of all 8 exercises to be completed within 60 min session. Progression: start with 1–2 sets and 10 repetitions (based on 80% one‐repetition maximum and on tolerance) with the aim of slowly and progressively increasing to 3 sets and 8–10 repetitions. *Reporting and Monitoring:* each patient completed an exercise diary after home exercise sessions. Weekly calls to increase motivation and assess rate of RPE. The 1‐repetition maximum was reassessed monthly, and the programme was adjusted accordingly. AE: Supervised aerobic exercise gym or hospital based 60 min twice per week Heart rate monitor and aerobic‐based home exercise programme provided. *Prescription*: warm‐up and cool‐down period of 5 min on a stationary exercise cycle, RPE of 11, followed by gentle stretching. Individually tailored on recumbent stationary exercise cycles, a treadmill, and elliptical trainer switching every 10 min, with 3 min rest. Intensity set to 80% heart rate reserve as derived from the incremental exercise testing with RPE training target was 13–15 (somewhat hard to hard). C: Control usual care group Seen routinely in the transplantation clinic but not referred for formal exercise.				

Randomized controlled trial of exercise in CKD—the RENEXC study				
Author, year	Hellberg *et al*., 2019^51^	Duration of intervention	12 months	
Participants CKD stage Age (years)	CKD—non‐dialysis Stages 3–5 66 ± 14 E1: 76 E2: 75	Sarcopenia outcomes		
		Muscle mass	Muscle strength	Physical performance
		NA	Between groups: ∆Grip strength (NS) ∆STS (NS) Within Group E1: ↑Grip strength (NS) ↑STS*** Within Group E2: ↔Grip strength (NS) ↑STS**	Between groups: ∆6‐MWT (NS) Within Group E1: ↑6‐MWT*** Within Group E2: ↑6‐MWT***
Intervention description				
Self‐administered exercise of 150 min/week distributed in 3–5 sessions/week starting with 10 min warm‐up followed by: 60 min of endurance training + 90 min/week of either strength or balance training. Both groups: Before starting, a bank of predefined exercises was created and explained in detail by the physiotherapist and individual training plan was provided. Training setting at home or at a nearby gym, depending on individual preference. Each patient was advised to evaluate training performance according to RPE and provide a report by mailing in the training diary. *Endurance training* to be performed for at least 60 min (2 sessions of 30 min)/week at an RPE of 13–15 includes walking, jogging, cycling, etc. and adjusted by increasing speed or distance, or by interval training. *Reporting and monitoring*: weekly phone calls by physiotherapist during first 3 months, followed by every second week in months 4 to 12, were provided to check progress, encourage patients, and adjust training plan to maintain desired level of exertion. E1: Strength + endurance training 90 min (3 sessions of 30 min)/week with a target of 13–17 RPE per exercise set. In all, 4–6 different exercises (e.g. quadriceps extension, squats, biceps curls, pull‐ups, etc.) were performed as 2–3 sets of 10 repetitions and adjusted by increasing the weights or the difficulty of the exercises (e.g. adjusting body position regarding angle or leverage). E2: Balance + endurance training 90 min (3 sessions of 30 min)/week at 13–17 RPE per exercise set. In all, 4–6 different exercises (e.g. standing with feet together, on one leg, on balance board or planking, etc.) were performed as 2–3 sets of 10 repetitions and adjusted by increasing the difficulty (e.g. adding arm movements, closing eyes, or changing body position).				

Effects of home‐based exercise on pre‐dialysis chronic kidney disease patients: a randomized pilot and feasibility trial				
Author, year	Hiraki *et al*., 2017^59^	Duration of intervention	12 months	
Participants CKD stage Age (years)	CKD—non‐dialysis Stages 3–4 68.7 ± 6.8 E: 14 C: 14	Sarcopenia outcomes		
		Muscle mass	Muscle strength	Physical performance
		NA	↑Grip strength (17.0 ± 16.1%)*	NA
Intervention description				
E: Home‐based therapy of combined aerobic and resistance exercise Exercise instructions were given in first visit included AE: brisk walking for 30 min a day or completing 8000–10 000 steps per day. RE minimum 3×/week: upper limbs using handgrip‐strengthening device provided and mid‐level load exercises such as squats and calf raises for exercising the lower limbs (20–30 repetitions per exercise). *Record keeping:* exercise record sheet used to report adherence to RE, the implementation rate and exercise details. Number of steps (steps/day), amount of exercise performed (total amount of calories burnt through exercise: kcal/day), and time spent on performing mid‐level load exercises (min/day) were collected from accelerometer pedometers worn continuously for 1 year and removed only when bathing or sleeping. No direct supervision for a period of 1 year. Exercises performed were collected from patients during outpatient visits every 2–3 months, and feedback was provided at each visit. Both the AE and the RE used RPE scale aiming to mid‐level load strength. C: Control group Given accelerometer pedometers for a period of 1 year. No exercise instructions were given and asked to carry out daily activities as usual. *Record keeping:* number of steps recorded during follow‐up visits, without additional information related to exercise.				

Exercise training in CKD: efficacy, adherence, and safety				
Author, year	Howden *et al*., 2015^60^	Duration of intervention	8 weeks supervised followed by 10 months home‐based exercise	
Participants CKD stage Age (years)	CKD—non‐dialysis Stages 3–4 E: 36 60.2 ± 9.7 C:36 62.0 ± 8.4	Sarcopenia outcomes		
		Muscle mass	Muscle strength	Physical performance
		NA	At 6 months follow‐up: ↔Grip strength* At 12 months Follow‐up: ↔Grip strength*	At 6 months follow‐up: ↑6‐MWT* ↔TUG (NS) (Prevented decline observed in control group) At 12 months follow‐up: ↑6‐MWT*** ↔TUG (NS) (Prevented decline observed in control group)
Intervention description				
E: Exercise training and lifestyle intervention Usual care plus assistance from a multidisciplinary team (nurse practitioner, exercise physiologist, dietitian, psychologist, diabetes educator, and social worker). Education about safety, hydration and signs and symptoms of abnormal response to exercise especially those with angina, severe arthritis, and diabetes was given. Individualized prescription aimed to complete 150 min/week of moderate intensity AE and RE, starting with 8 weeks of supervised training followed by 10 months of home‐based training. *Initial supervised phase:* AE for 30 min: walking or jogging, cycling, or rowing at an exercise intensity of RPE of 13–15. RE: 3 sets/10–15 repetitions of 6–8 functional RE, using hand weights or resistant bands focusing on whole‐body, including wall squats; bench press; lunges; wall push‐ups; seated row, bicep, and triceps extension; ‘supermans’; and bridge holds. Follow‐up with the nurse practitioner was scheduled at Week 4. *Home‐based maintenance phase:* Encouragement to continue to perform a combination of AE and RE. All participants were provided with resistance bands, Swiss ball, and RE booklet with examples of strength training workouts. Home‐based AE consisted predominantly of walking or stationary cycling. Regular contact to monitor adherence to training. If issues identified with adherence, they were encouraged to attend the gym for a refresher visit or alternative strategies were discussed to reach the required exercise levels. C: Control group Received usual care according to best practice guidelines including attending a consultation with a nephrologist and lifestyle modification recommendation with no detailed information, education, or referral to an allied health practitioner.				

Anabolic exercise in haemodialysis patients: a randomized controlled pilot study				
Author, year	Kirkman *et al*., 2014^58^	Duration of intervention	12 weeks	
Participants CKD stage Age (years)	ESRD on haemodialysis RE: 9 48 ± 18 Sham E:10 58 ± 15	Sarcopenia outcomes		
		Muscle mass	Muscle strength	Physical performance
		↑ASMM by MRI**	↑STS (NS)	↑6‐MWT (NS) ↓TUG (NS)
Intervention description				
3×/week during haemodialysis E: progressive resistance exercise training (PRET) Each session: included leg press exercise using equipment fit to dialysis chair with series of resistance bands providing a maximum resistance equivalent to 200 kg. Exercise included 3 sets/8–10 repetitions with 2 min rest between sets at 80% of patients predicted 1‐RM. If 10–12 repetitions could be completed at a rating of RPE below 15 (hard), 1‐RM was re‐determined and the training load increased accordingly. Weekly training volume was calculated as kg per lift × lifts per session × sessions per week. SHAM E group: Un‐progressive stretches using an ultra‐light band.				

Effect of resistance exercises on the indicators of muscle reserves and handgrip strength in adult patients on haemodialysis				
Author, year	Olvera‐Soto *et al*., 2015^54^	Duration of intervention	12 weeks (24 sessions in total)	
Participants CKD stage Age (years)	ESRD on haemodialysis 29 (21–39) E: 30 C: 31	Sarcopenia outcomes		
		Muscle mass	Muscle strength	Physical performance
		NA	↑Grip strength (9.82%)**	NA
Intervention description				
2×/week during haemodialysis E: Progressive resistance exercise First 2 sessions; familiarization with exercises. 3rd session; 500‐g weight belts were attached to each ankle 4 sets/30 repetitions were performed for each 4 exercises: A: arm extension with moderate resistance bands in the non‐arteriovenous fistula arm whereas patients with catheters both arms exercised B: lower leg extension C: straight leg extension D: seated marching Duration per session: 50 min. C: Control group No exercises, education, or equipment to perform resistance exercises or any type of exercises were given.				

Effect of continuous progressive resistance training during haemodialysis on body composition, physical function and quality of life in end‐stage renal disease patients: a randomized controlled trial				
Author, year	Rosa *et al*., 2018^55^	Duration of intervention	12 weeks	
Participants CKD stage Age (years)	ESRD on haemodialysis 55.7 ± 14.03 E: 28 C:24	Sarcopenia outcomes		
		Muscle mass	Muscle strength	Physical performance
		Between groups: ∆ASMM by DXA* Within group (E): ↑ASMM by DXA*	Between groups: ∆Grip strength (NS) ∆STS* Within group (E): ↔Grip strength (NS) ↑STS*	Between groups: ∆6‐MWT (NS) Within group (E): ↑6‐MWT*
Intervention description				
3×/week with each session divided to 2 segments; Upper limb exercises prior to haemodialysis in waiting room and lower limb exercises during haemodialysis. Clinical exercise physiologist supervised all exercise sessions in both groups. E: Progressive resistance exercise 6 sessions of familiarization exercises held 2 weeks prior to training with no/low loads at 2 sets/10 repetitions. Post 2 weeks: start 2 sets of 15–20 repetitions of 11 exercises progressively increased until momentary failure occurred. If repetitions performed beyond the above, weight was increased to return the number of repetitions within the maximum training zone (15–12 repetitions). Rest between sets and exercises was individualized according to patients' needs. Post exercise; passive stretching of lower limbs performed to facilitate recovery. Duration per session: 40–50 min. C: Sham exercise Active mobilization of the arms and legs without load and progression, circumduction of the cervical and scapular girdle, and a breathing exercise. 2 sets of 3–5 repetitions only and no stretching exercises. Duration per session 5–10 min.				

Effects of a renal rehabilitation exercise programme in patients with CKD: a randomized, controlled trial				
Author, year	Rossi *et al*., 2014^61^	Duration of intervention	12 Weeks (24 sessions)	
Participants CKD stage Age (years)	CKD—non‐dialysis Stages 3–4 E: 48 67.7 ± 12.4 C: 46 69.2 ± 12.4	Sarcopenia outcomes		
		Muscle mass	Muscle strength	Physical performance
		NA	↑STS (29%)***	↑6MWT (19%)*** ↔Gait speed (NS)
Intervention description				
2×/week at selected physical therapy or cardiac rehabilitation facilities E: renal rehabilitation exercise programme (RRE) 60 min individual or group sessions guided by exercise physiologist or physical therapist who assessed cardiovascular and strength capabilities at the initial session according to the perceived level of exertion (PLE) scale. Intervention limited to PLE 11 corresponding to 60–65% predicted maximal heart rate. *Cardiovascular exercises (AE):* Treadmill walking and/or stationary cycling with increase of duration by 2–3 min/session, increase bicycle freewheel tension or treadmill speed or elevation. Self‐administered 5000–10 000 steps/day monitored by pedometers. *Weight training (RE):* Upper and lower extremity extensions and flexions with free weights. 1 set/10 repetitions of each exercise using 1 to 10 lb weights (according to tolerance) and increased to 3 sets of 15 repetitions, after which time weight was further increased. C: Control group Received usual care.				

Effects of progressive resistance training on body composition, physical fitness and quality of life of patients on haemodialysis				
Author, year	Song *et al*., 2012^56^	Duration of intervention	12 weeks	
Participants CKD stage Age (years)	ESRD on haemodialysis E: 20 52.1 ± 12.4 C: 20 54.6 ± 10.1	Sarcopenia outcomes		
		Muscle mass	Muscle strength	Physical performance
		↑SMM by BIA**	↑Grip strength (NS) ↑STS (NS)	NA
Intervention description				
3×/week during haemodialysis E: Progressive resistance training (PRT) 3 sets of 10–15 repetition with intensity of 11–15 RPE (‘moderate’ to ‘hard’). 10 PRT exercises in 30 min/PRT session including 5 min warm up prior to PRT and 5 min cool down post PRT. 20 min PRT included 6 upper body exercises using elastic bands and 6 lower body exercises using sand bags. Elastic bands tensile strength was progressively increased to all participants. 4th week of PRT, 1–3 kg sand bags added around each of ankles. 8th week of PRT, +1 kg was added to previous sand bag worn. C: Control group Usual care without any instructions to exercises or access to exercise equipment.				

Muscle mass and plasma myostatin after exercise training: a substudy of Renal Exercise (RENEXC)—a randomized controlled trial				
Author, year	Zhou *et al*., 2019^50^	Duration of intervention	12 months	
Participants CKD stage Age (years)	CKD—non‐dialysis Stages 3–5 67 ± 13 E1: 53 E2: 59	Sarcopenia outcomes		
		Muscle mass	Muscle strength	Physical performance
		Between groups: ∆ SMM by DXA (NS) ∆ ASMM by DXA (NS) Within Group (E1): ↔SMM by DXA* ↔ASMM by DXA* Within group (E2): ↑SMM by DXA** ↑ASMM by DXA*	NA	NA
Intervention description				
Refer to intervention details described in Hellberg *et al*.^51^ above.				

Data shown as either Mean ± SD or Median (Range); CKD: chronic kidney disease; RE: resistance exercise; AE: aerobic exercise; ET: endurance training; E: exercise; C: control; RPE: rated perceived exertion; 1‐RM: one repetition maximum; SMM: skeletal muscle mass; ASMM; appendicular skeletal muscle mass; BIA: bioelectrical impedance analysis; MRI: magnetic resonance imaging; DXA: dual‐energy absorptiometry; STS: sit‐to‐stand test; TUG: timed‐up‐and‐go test; 6MWT: 6 min walking test; SPPB: short performance physical battery; NA: not available.

∆, change; ↑, increase, ↓, decrease; ↔, no change. NS, not significant.

* *P* < 0.05.

** *P* < 0.01.

*** *P* < 0.001.

## Appendix 2 Clinical studies of resistance exercise‐based interventions and nutritional supplementation and their effects on sarcopenia outcomes in CKD

**Table:**

Effect of resistance exercise plus cholecalciferol on nutritional status indicators in adults with Stage 4 chronic kidney disease				
Author, year	Olvera‐Soto *et al*., 2019^68^	Duration of intervention	12 weeks	
Participants CKD stage Age (years)	CKD—non‐dialysis Stage 4 48 (36–52) I: 26 C: 13	Sarcopenia outcomes		
		Muscle mass	Muscle strength	Physical performance
		Between groups: %∆ SMM by BIA (NS) Within group (intervention): ↑SMM by BIA (NS)	Between groups: %∆ Grip strength (right hand)* %∆ Grip strength (left hand)* Within group (intervention): ↑Grip strength (right hand)*** ↑Grip strength (left hand)**	NA
Intervention description				
I: Intervention group: resistance training 60 min ×3/week + daily oral cholecalciferol supplementation. *Resistance exercise programme;* start: 15 min warm‐up period, next: light or medium resistance bands used to perform each of the 6 exercises with 8 repetitions of each: scapular retraction, scapular protraction, scapular depression with shoulder abduction, elbow flexion, shoulder abduction, sit‐ups. Record keeping: patients received training to fill a log to report days, duration, and intensity of exercise sessions. *Cholecalciferol intake:* dosing scheme according to serum levels: serum concentration >20 ng/dL; supplement with 600 IU cholecalciferol/day, serum concentration 10–19.9 ng/dL; supplement with 1600 IU cholecalciferol/day, serum concentration <10 ng/dL; supplement with 7200 IU cholecalciferol/day. Record keeping: patients received training to fill a log to report days, frequency, and dosage of cholecalciferol intake. Control group: standard medical care without participation in exercise programme.				
Effect of oral nutritional supplementation with and without exercise on nutritional status and physical function of adult haemodialysis patients: a parallel controlled clinical trial (AVANTE‐HEMO Study)				
Author, year	Martin‐Alemany *et al*., 2019^72^	Duration of intervention	12 weeks	
Participants CKD stage Age (years)	ESRD on haemodialysis (2×/week) 29 ± 9.3 (1) ONS: 13 (2) ONS + RE: 9 (3) ONS + AE: 12	Sarcopenia outcomes		
		Muscle mass	Muscle strength	Physical performance
		NA	Within group (ONS): ↑Grip strength* ↑STS (NS) Within group (ONS + RE): ↑Grip strength* ↑STS* Within group (ONS + AE): ↑Grip strength* ↑STS* Effect size (Cohen's *d*): *Grip strength* ONS + RE (1.01) ONS + AE (0.60) ONS (0.11) *STS* ONS + RE (0.81) ONS + AE (1.20) ONS (0.52)	Within group (ONS): ↓TUG* ↑6MWT* Within group (ONS + RE): ↓TUG* ↑6MWT* Within group (ONS + AE): ↓TUG* ↑6MWT* Effect size (Cohen's *d*): *TUG* ONS + RE (1.04) ONS + AE (1.6) ONS (0.91) *6MWT* ONS + RE (0.94) ONS + AE (1.11) ONS (0.35)
Intervention description				
All patients were provided with a 35 kcal/kg diet plan adjusted for age, sex, and physical activity and consists of: 1.2 g protein/kg, 25–35% fat, and 50–60% carbohydrates as percentages of the total energy requirement. Oral nutritional supplementation group (ONS): during haemodialysis sessions ×2/week 1 can of specialized ONS for maintenance dialysis. Each can consist of 480 kcal, 20 g protein, 20 g lipids, and 56 g carbohydrates. Content includes water, maltodextrin, canola oil, lactalbumin, ascorbic acid, and citric acid as antioxidant. ONS plus aerobic exercise group (ONS + AE): during haemodialysis sessions ×2/week *ONS:* ½ can of the specialized formula described above during 1st hour of haemodialysis session; other ½ of the can administered after AE routine. *AE:* 20–30 min pedalling stationary in the first 2 h of haemodialysis with aim of moderate intensity (12–13 RPE). ONS plus resistance exercise group (ONS + RE): during haemodialysis sessions ×2/week *ONS:* ½ can of the specialized formula described above during 1st hour of haemodialysis session; other ½ of the can administered after RE routine. RE: 40 min of 4 types of exercise using resistance bands performed in the first 2 h of haemodialysis (4 sets/20 repetitions) with aim of moderate intensity (12–13 RPE). Both exercise groups: RE and AE was supervised by a trained dietitian with experience in exercise programmes for dialysis patients. Weight, resistance of the bands in RE, time of AE, and resistance of the bicycles were increased when the patient's RPE was less than the target.				

The effect of resistance exercise to augment long‐term benefits of intradialytic oral nutritional supplementation in chronic haemodialysis patients				
Author, year	Dong *et al*., 2011^69^	Duration of intervention	6 months	
Participants CKD stage Age (years)	ESRD on haemodialysis 43 ± 13 ONS: 12 ONS + RE:10	Sarcopenia outcomes		
		Muscle mass	Muscle strength	Physical performance
		Between groups at 3, 6 months: ∆ SMM (kg) by DXA (NS) ∆ ASMM (kg) by DXA (NS)	NA	NA
Intervention description				
ONS group: Received 2 cans ONS within 30 min prior to dialysis session 3×/week. Each supplement dose (2 cans) contained 480 mL, 960 Kcal (132.8 kcal from protein, 412.8 kcal from carbohydrates, and 412.8 kcal from fat). Weekly visits were completed by study personnel with each subject to evaluate tolerance and compliance to the supplement and to restock additional supplement. ONS + RE group: *ONS* Received same intervention as ONS only group. *RE* Supervised 3 sets of 12 repetitions of leg‐press using a specialized machine to target quadriceps, hamstring, and gluteus muscles within 30 min prior to dialysis session 3×/week. Participants sat on the leg press machine with feet placed on a platform, legs at a 90‐degree angle, and instructed to push the platform forward, leaving knees slightly bent. Individualized exercise intensity: first month set at 70% of each participant's 1 RM using weight equal to participant's body weight Additional weight (~25–50 lb) added at each repetition until temporary muscle failure. At the month 3 and month 6 assessments, 1‐RM was repeated in all participants to evaluate progress and determine a new 1‐RM.				

The effects of high‐load strength training with protein‐containing or non‐protein‐containing nutritional supplementation in patients undergoing dialysis				
Author, year	Molsted *et al*., 2013^70^	Duration of intervention	16 weeks	
Participants CKD stage Age (years)	ESRD on haemodialysis and peritoneal dialysis 55 ± 14 E + PRO: 16 E + Non‐PRO: 13	Sarcopenia outcomes		
		Muscle mass	Muscle strength	Physical performance
		NA	All participants: ↑STS*** (between control period and training period) Diff between the two groups: STS (NS)	NA
Intervention description				
Participants were recruited to a control period of 16 weeks with no intervention followed by an intervention period of 16 weeks with strength training. Progressive high‐load strength training in both groups 3×/week outside of dialysis supervised by physiotherapists and exercise instructors. Participants chose which 3 days they exercised with advice to spread out sessions during the week; if necessary, exercising 2 consecutive days was allowed, to comply with dialysis schedule. Warm‐up and exercise: 5 min of stationary ergometer followed by leg press, leg extension, and leg curl. Rest period between each set of exercises: 60–90 s. Number of exercise repetitions to be completed until muscle exhaustion with load progressively increased according to changes in 1 repetition maximum (tested and adjusted 6 times during study period). Supplementations in both groups: Both contained a low amount of potassium and phosphate as recommended to patients undergoing dialysis. The participants were instructed not to ingest meals at least 2 hours on either side of the training sessions. PRO group: Provided with protein supplementation of 125 mL containing 9.4 g protein (100% whey, 14.3% leucine), 25 g carbohydrate, and 12.5 g lipid (250 kcal). The non‐PRO group: Provided with energy supply of 54.5 mL containing 2.4 g carbohydrate and 27.3 g lipid (250 kcal).				

The effects of resistance exercise and oral nutritional supplementation during haemodialysis on indicators of nutritional status and quality of life				
Author, year	Martin‐Alemañy *et al*., 2016^71^	Duration of intervention	3 months	
Participants CKD stage Age (years)	ESRD on haemodialysis (2×/week) 34 (24.5–43) ONS plus RE: 17 Control ONS: 19	Sarcopenia outcomes		
		Muscle mass	Muscle strength	Physical performance
		NA	Within group (ONS): ↑Grip strength* Within Group (ONS + RE): ↑Grip strength*	NA
Intervention description				
ONS both groups Consisted of 434 kcal, 19.2 g protein and 22.8 g lipids, low in vitamins A and D and high in folates and vitamin B6, with high‐oleic safflower oil, corn syrup solids, and fructooligosaccharides (FOSs). ONS plus RE *ONS* Oral nutritional supplement given during dialysis, with ½ a can administered during the 1st hour of the haemodialysis session and ½ a can administered after the RE routine *RE* RE performed once/week with total of 24 sessions of RE at the end of study during the 2nd hour of haemodialysis session (four sets of 30 repetitions for 40 min). 1 week prior to start of clinical study, patients performed physical conditioning exercise without any extra weight 500 g ankle weights and medium‐resistance springs for hands and arms used in RE with adjustment according to location of the vascular access. Four types of RE used: lower leg extension; arm extension with medium resistance springs leg raises from semi‐recumbent position; knees‐bends to chest Each exercise lasted 10 min separated by 3 min of rest. The patients were advised to work with a level of perceived exertion of ‘somewhat strong’ (12–13 RPE) Control group (ONS) During haemodialysis sessions, patients received a can of a specialized ONS for maintenance dialysis patients				

Data shown as mean ± *SD* or median (range). 1‐RM, one repetition maximum; 6MWT, 6 min walking test; AE, aerobic exercise; ASMM, appendicular skeletal muscle mass; C, control; CKD, chronic kidney disease; DXA, dual‐energy absorptiometry; E, exercise; I, intervention; NA, not available; ONS, oral nutritional supplementation; RE, resistance exercise; RPE, rated perceived exertion; SMM, skeletal muscle mass; STS, sit‐to‐stand test; TUG, timed‐up‐and‐go test.

∆, change; %∆, per cent change; ↑, increase; ↓, decrease; ↔, no change. NS, not significant.

* *P* < 0.05.

** *P* < 0.01.

*** *P* < 0.001.
